# Fluorescence Quenching-Based Mechanism for Determination of Hypochlorite by Coumarin-Derived Sensors

**DOI:** 10.3390/ijms20020281

**Published:** 2019-01-11

**Authors:** Karolina Starzak, Arkadiusz Matwijczuk, Bernadette Creaven, Alicja Matwijczuk, Sławomir Wybraniec, Dariusz Karcz

**Affiliations:** 1 Department of Analytical Chemistry (C1), Faculty of Chemical Engineering and Technology, Cracow University of Technology, Warszawska 24, 31-155 Cracow, Poland; kstarzak@chemia.pk.edu.pl (K.S.); swybran@chemia.pk.edu.pl (S.W.); 2 Department of Biophysics, University of Life Sciences in Lublin, Akademicka 13, 20-950 Lublin, Poland; arkadiusz.matwijczuk@up.lublin.pl (A.M.); alicja.matwijczuk@up.lublin.pl (A.M.); 3 Centre of Applied Science for Health, Institute of Technology Tallaght, Dublin 24, Ireland; Bernie.Creaven@it-tallaght.ie

**Keywords:** coumarin, hypochlorite sensing, fluorescence quenching

## Abstract

A fluorescence quenching-based mechanism for the determination of hypochlorite was proposed based on spectroscopic and chromatographic studies on the hypochlorite-sensing potency of three structurally similar and highly fluorescent coumarins. The mode of action was found to rely upon a chlorination of the coumarin-based probes resulting from their reaction with sodium hypochlorite. Importantly, the formation of chlorinated derivatives was accompanied by a linear decrease in the fluorescence intensities of the probes tested. The results obtained suggest the applicability of a coumarin-dependent hypochlorite recognition mechanism for the detection of, as well as for quantitative determination of, hypochlorite species in vitro.

## 1. Introduction

The research on coumarin-based sensors is well-established and countless coumarin derivatives have been reported as potential tools for the detection and recognition of various chemical species [1,2,3,4]. Regardless of the abundant literature on the subject, a large number of processes governing the modes of action of coumarin-based fluorescent probes remain unclear. The majority of reports discuss the photophysical aspects of these mechanisms based on photO^−^induced electron transfer (PET), intramolecular charge transfer (ICT), fluorescence resonance energy transfer (FRET), or excited state intramolecular proton transfer (ESIPT) phenomena [5,6]. Other reports refer to various fluorescence quenching mechanisms as essential for signalling [6,7]. Chemically, these processes may involve multistep reactions and, in many cases, lead to the formation of diverse products, where identification may prove difficult.

The interest of our group in coumarins is primarily due to their significant antimicrobial activities enhanced upon their complexation with selected transition metal salts. Until now, our investigations have resulted in a synthesis of Cu(II) and Ag(I) complexes incorporating various coumarin-derived ligands [8,9,10,11,12,13,14]. Most of the obtained complexes demonstrated high antimicrobial and antifungal activities and numerous aspects of their therapeutic modes of action were studied by our group in detail [15,16,17]. 

Our current interest in coumarins extends to the possible application of highly fluorescent coumarins as probes for the detection of reactive oxygen species (ROS) and particularly the hypochlorite ion (ClO^−^). In more detail, the presence of ClO^−^ in mammalian tissue, which is generated from the reaction of hydrogen peroxide with ubiquitous chloride ions, has been shown to result in oxidative damage to tissues and is associated with inflammation processes. Its presence in tissue can thus serve as an inflammation marker. Our previous studies revealed an ability of naturally occurring betalain pigments to effectively scavenge the toxic ClO^−^ species from in vitro inflammation-mimicking systems [18,19]. These results prompted us to search for hypochlorite-specific fluorescent probes, which would allow for the determination of ClO^−^ in such systems. Investigations into various aspects of the antioxidative activity of betalains [18,19,20,21,22,23,24] and photophysical properties of 1,3,4-thiadiazole derivatives [25,26,27,28,29,30,31,32,33] have now extended to coumarin compounds, whose fluorescent properties have been noted by many groups. 

The comparison of recognition mechanisms for detecting ClO^−^ suggests that the key step relies upon the oxidation of a specific moiety attached to the main fluorophore and that the oxidative action of ClO^−^ results either in fluorescence quenching or in a significant shift in the fluorescence maximum of the probe. ClO^−^ detection by coumarin-based probes has been reported to occur in a similar manner [34]. The chemistry which governs this mechanism is seemingly simple, although some cases may remain ambiguous, and various factors such as the probe concentration, sample environment, and light scattering have to be considered [35]. Also, the structural elucidation of the oxidation products formed has not been studied in detail. 

**Figure 1:**
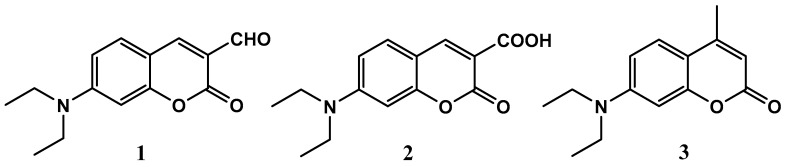
Structural formulae of fluorescent coumarins used for the in vitro detection of hypochlorite ions: (**1**) 7-diethylamino-3-formylcoumarin, (**2**) 7-diethylaminocoumarin-3-carboxylic acid, and (**3**) 7-diethylamino-4-methylcoumarin.

In this context, in the detection of hypochlorite via a coumarin-derived probe, 7-diethylaminocoumarin-3-carboxylic acid **2** was reported as the main product formed from the oxidative reaction of hypochlorite with 7-diethylamino-3-formylcoumarin **1**, resulting in a significant decrease in the fluorescence intensity associated with **1** (Figure 1) [34]. On the other hand, compound **2** is well known for its highly intensive fluorescence emission [6,36,37]. The formation of a fluorescence product would mean that the use of fluorescence quenching as the basis of quantification of hypochlorite would be necessarily problematic. This conundrum prompted our group to a more in-depth investigation into the potential possibility of the quantitative determination of ClO^−^ content based on an investigation into hypochlorite′s interactions with a number of highly fluorescent coumarin derivatives and the identification of the oxidation products formed. Careful identification of the derivatives formed upon ClO^−^ recognition/detection and their stoichiometry may result in the design of new methods for the quantitative determination of ClO^−^ ions and may open a new chapter in coumarin-based sensors as well as in antioxidative agent chemistry. 

## 2. Results and Discussion

Three coumarin derivatives, namely 7-diethylamino-3-formylcoumarin **1**, 7-diethylaminocoumarin-3-carboxylic acid **2**, and 7-diethylamino-4-methylcoumarin **3** (Figure 1), were chosen as models. Their selection was primarily dictated by the presence of the 7-diethylamino group, which together with the 3-substituted coumarin lactone ring constitute a structural motif present in a large number of highly fluorescent coumarins [38] and coumarin-derived sensors. The substituents present at the lactone ring, namely the formyl and carboxyl groups in **1** and **2**, respectively, were selected in accordance with the previously proposed mechanism of ClO^−^ detection by coumarin-based probes [34]. The selection of **3** was dictated by its well-known and highly intensive fluorescence as well as the lack of highly reactive substituents at its lactone ring.

### 2.1. Determination of the Effect of Hypochlorite on the Emission Properties of the Probes

The excitation of **1** at λ_Ex_ 289 nm results in an intensive emission with a maximum at λ_Em_ 464 nm. Based on the previously reported recognition mechanism for detecting ClO^−^ species by the oxidation of **1**, the addition of increasing concentrations of hypochlorite into the solution of **1** results in the linear decay of the fluorescence emission, and the reaction is easily monitored by spectrofluorimetry [34]. It has been assumed that the formation of **2** is the main reason for the fluorescence drop. On the other hand, the excitation of **2** at λ_Ex_ 289 nm results in highly intensive fluorescence (λ_Em_ 460 nm). This in turn allows the assumption that the interaction of ClO^−^ with **1** may lead to the formation of another product (products), which is (are) structurally different from **2** and these structural differences may be responsible for the fluorescence drop observed. Taking into account the reactive and oxidation-prone characteristics of the formyl group, the initial formation of 3-carboxylic acid **2** is likely. However, decarboxylation and further chemical reactions cannot be excluded. In order to verify this hypothesis, both **1** and **2** were investigated for their ability to react with ClO^−^ species using spectrofluorimetric monitoring of these interactions. Moreover, a series of LC-MS experiments were performed in order to identify the reaction products and assess the possibility of the determination of ClO^−^ based on the quantitative determination of the coumarin derivatives formed. In addition, a commercially available fluorescence standard, namely 7-diethylamino-4-methylcoumarin (Coumarin II) **3** was investigated in a similar manner. Due to its structural similarity to both **1** and **2**, compound **3** demonstrates similar fluorescence properties (λ_Ex_ 289 nm, λ_Em_ 450 nm), but it lacks the carbonyl substituents at its lactone ring. 

**Figure 2:**
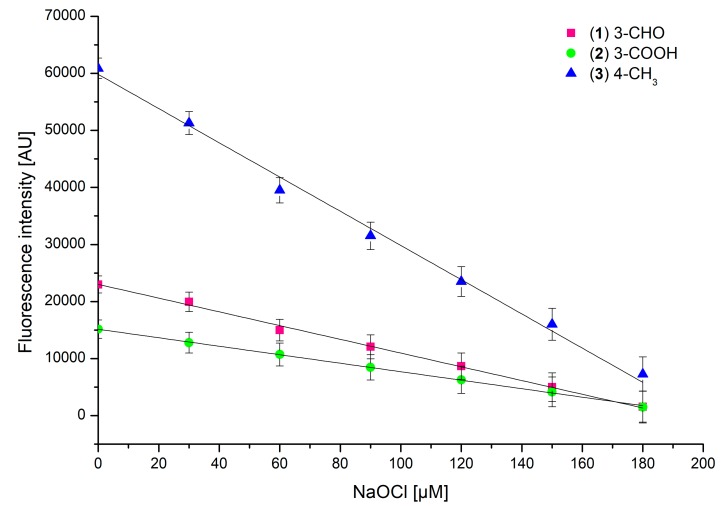
Changes in fluorescence intensity of 150 µM of **1**–**3** induced by an increase in NaOCl concentration, at pH 5, λ_Ex_ 289 nm (λ_Em_ 464 nm, 460 nm, and 450 nm, respectively), and a temperature of 25 °C.

The potential practical application of **1**–**3** as fluorescent probes for detecting ClO^−^ species in biological samples necessitates an assessment of their reactivity with various concentrations of sodium hypochlorite. Due to the high physiological concentration of Cl^−^ (100–140 mM), more than 80% of the H_2_O_2_ generated is used to form 20–400 μM HOCl per hour [39]. Thus, the average concentration of Cl^−^ ions in saline has been reported to oscillate around 150 μM. Considering the fact that chlorine ions are known to play a key role as substrates in the enzymatic synthesis of hypochloric acid [40,41] and assuming that the coumarins **1**–**3** react with hypochlorite with 1:1 stoichiometry, the concentration of each coumarin derivative during the testing was 150 μM. The first set of experiments involved a comparison of the fluorescence emissions of **1**–**3** upon the addition of increasing hypochlorite aliquots. The measurements were carried out under identical conditions, namely the excitation wavelength λ_Ex_ 289 nm, pH 5, and a temperature of 25 °C. All coumarin derivatives demonstrated a notable decrease in their fluorescence intensities upon an increase in the sodium hypochlorite concentration, with almost complete fluorescence quenching at 180 μM of hypochlorite (Figure 2).

**Figure 3:**
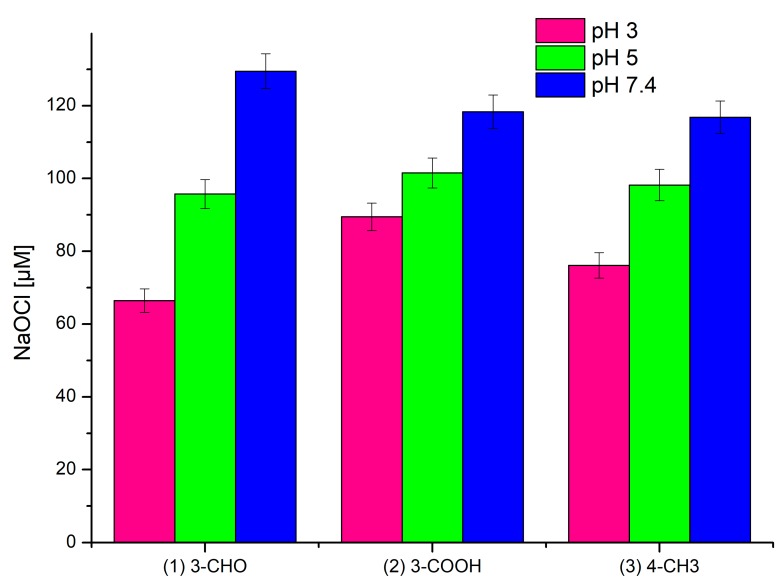
NaOCl concentration causing a 50% decrease in the fluorescence intensity of 150 µM of probes **1**–**3** at various pH levels, λ_Ex_ 289 (λ_Em_ 464 nm, 460 nm, and 450 nm, respectively), and a temperature of 25 °C.

Compared to those of **1** and **2**, the fluorescence intensity drop in **3** was the most significant. In all of the studied compounds, the fluorescence intensity changes had a linear characteristic, suggesting a directly proportional dependence of fluorescence intensity on the sodium hypochlorite concentration. Concentrations of hypochlorite causing a 50% decrease in fluorescence intensity of 150 μM solutions of **1**–**3** are given in Figure 3.

### 2.2. The pH Effects on the Hypochlorite Detection by Coumarin-Derived Probes

It is well-known that the pH in the body varies from highly acidic to slightly basic. For instance, the initial part of the gastrointestinal tract in humans, and especially the stomach cavity, is characteristically of strong acidity. Active phagosomes in neutrophils are normally at pH 5 where the endogenic hypochlorous acid is formed, while body fluids such as blood or saliva demonstrate typical pH values up to 7.4 [42,43]. In order to imitate the conditions occurring in various parts of the organism, testing was carried out at three pH values, namely at 3, 5, and 7.4.

The measurements carried out at the pH levels of 3 and 7.4 gave results almost identical to those obtained at pH 5. At pH 3, the hypochlorite concentration equivalent to a fluorescence quench was only slightly lower, while at pH 7.4 it was higher than 180 μM (see supplementary data).

The subtle differences in the reactivity of **1**–**3** towards sodium hypochlorite at various pH levels may result from the pH profile for reactive chlorine species, which states that the dissociation of sodium hypochlorite in aqueous solution varies depending on the pH. An acidic pH favours the formation of both hypochlorous acid (HClO) and molecular chlorine (Cl_2_), while at a basic pH the hypochlorite ion (ClO^−^) is predominant [43]. At a low pH, the strong oxidative properties of hypochlorous acid may favour the oxidation of the carbonyl group in **1** with the formation of the carboxyl derivative **2** and its subsequent decarboxylation. Secondly, the presence of Cl_2_ species may lead to the formation of chlorinated derivatives. Neither the oxidation of **1** nor the decarboxylation of **2** is favoured at a high pH, although the chlorination may occur as result of ClO^−^ action. Thus, the profile of products of the reaction of hypochlorite at different pH levels should be a reflection of the reaction of the dominant species at each pH value, and the differences observed in Figure 3 correlate with the LC-MS data (see Section 2.3). Therefore, the formation of chlorinated derivatives may be a pH-independent process responsible for the fluorescence quenching in coumarin derivatives **1**–**3**.

### 2.3. Mass Spectrometry Analysis of the Reaction Mixtures 

In order to verify the possibility of the formation of chlorinated derivatives, a number of HPLC-PDA-ESI-MS analyses were carried out. Two series of samples were analysed. In the first series, equimolar amounts of sodium hypochlorite and probes **1**–**3** were buffered at pH 3, 5, and 7.4, respectively, prior to analysis. A second series of samples was prepared in a similar manner, except that the probe:NaOCl ratio was 1:5 (n/n). Sodium hypochlorite-free solutions of **1**–**3** were used as a reference. The results obtained for first series at pH 3 are given in Table 1. The data recorded at pH 5 and 7.4 was identical except that no dichloro-substituted derivatives were detected (see supplementary data).

**Figure 4:**
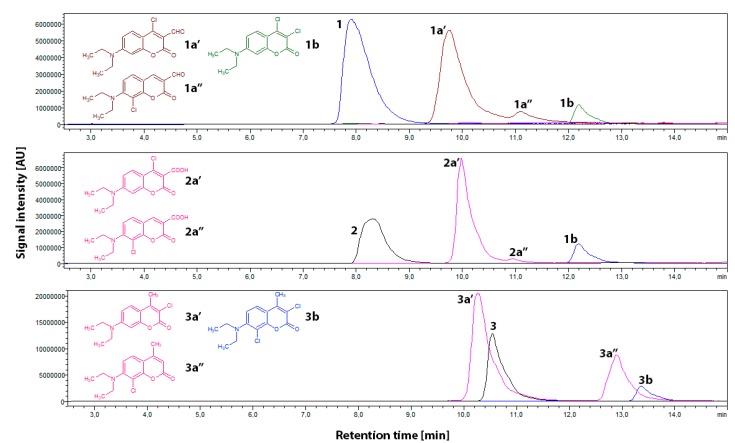
HPLC-MS traces and proposed structures of chlorination products of 150 μM probes **1**–**3** by 150 μM of NaOCl at pH 3 and a temperature of 25 °C. Peak numbering is presented in Table 1. (The remaining HPLC-MS data are given in the supplementary information).

The first series of HPLC-PDA-ESI-MS experiments was carried out on samples with a ratio of 1:1 (probe:NaOCl). Compared to the reference samples, the data obtained revealed that in all cases a substantial amount of unreacted probe was detected. Secondly, each sample showed that the dominant product had a characteristic *m*/*z* value higher by 34 amu compared to that of the respective probes **1**–**3**. This correlates with the formation of monochloro-substituted derivatives, namely **1a′** (*m*/*z* 280 protonated molecular ion), **2a′** (*m*/*z* 296), and **3a′** (*m*/*z* 266). The *m*/*z* values corresponding to the ^37^Cl isotope were present in correct ratios. A more careful study of the LC-MS data revealed that the main products **1a′**, **2a′**, and **3a′** are accompanied with their respective isomers **1a′′**, **2a′′**, and **3a′′**, differing from one another in polarity and their UV-Vis absorption maxima (Table 1). Compared to that of **1a′** and **2a′**, the respective amount of **1a′′** and **2a′′** was negligible, while the **3a′**: **3a′′** ratio was approximately 2:1 (Figure 4). There was no evidence for the formation of any other monochloro-substituted derivatives. It is particularly worth emphasizing that the analyses of samples containing **1** gave no evidence of the formation of **2** or any other carboxylic acid derivative of **1**. Interestingly, the hypochlorite mixtures either with **1** or **2** revealed the presence of a new signal characteristic of *m*/*z* 286 amu. Regardless of the difference of the substituent at the C3 position of the probe (formyl group in **1** and carboxyl group in **2**), the comparison of chromatographic, spectrophotometric, and mass-spectrometric data confirmed the formation of identical products. This common derivative was assigned as dichloro-substituted 7-diethylaminocoumarin **1b**. This, in case of probe **1**, suggests the oxidation of the formyl moiety and subsequent decarboxylation/chlorination at the C3 carbon. A similar decarboxylation/chlorination effect was observed in probe **2**, except that the oxidation step was not considered, since probe **2** already has the carboxyl group at its C3 carbon. The formation of **1b** was even more pronounced upon an increase in hypochlorite concentration (see paragraphs below). In samples containing compound **3**, which lacks the carbonyl substituent, a signal at *m*/*z* value 300 amu was detected, corresponding with the formation of dichloro-7-diethylamino-4-methylcoumarin **3b** (Figure 4, Table 1).

Although the LC-MS data do not allow for a direct determination of the position at which chlorination occurs, some estimations can be made. Taking into account that both **1** and **2** have their C3 positions occupied by carbonyl (carboxyl) groups, their chlorination may occur either at aromatic C8 and C6 or at C4 of the lactone ring. In case of compound **3**, the chlorination pattern would most likely occur in a similar manner as that in **1** and **2** except for the lactone ring, where the C3 position is available for chlorination. Since the chlorination of the aromatic ring occurs generally via electrophilic substitution, the chlorination at the C5 position is rather unlikely, due to an inductive withdrawal of electrons caused by the 7-diethylamine group. Moreover, based on a recent report [34], the use of the 6,8-disubstituted analogue of **1** does not interfere with the recognition mechanism proposed therein, which in light of our findings implicates that the chlorination occurs primarily at the lactone ring. Therefore, **1a′**, **2a′**, and **3a′** may be assigned as the respective monochloro derivatives of **1**, **2**, and **3** chlorinated at the lactone ring, while **1a′′**, **2a′′**, and **3a′′** may represent the respective isomers chlorinated at the aromatic ring (Table 1).

The second series of HPLC-PDA-ESI-MS experiments was carried out on samples containing ratios of 1:5 (probe:NaOCl). An excess of hypochlorite was used in order to achieve the complete conversion of **1**–**3** and to evaluate the possibility of di- and trichchloro-substituted derivatives as well as the formation of oxidation products of **1**–**3,** which did not form in the 1:1 (probe:NaOCl) mixtures. As expected, the results obtained showed no evidence for the presence of unreacted probes (**1**–**3**). Secondly, the monO^−^chlorinated derivatives **1a′**, **2a′**, and **3a′** remained dominant. The signal originating from **1b** (*m*/*z* 286) was about 30% more intensive than that in the 1:1 (probe:NaOCl) mixtures. Although the **1b** formation mechanism based on the oxidation and subsequent decarboxylation/chlorination seems valid at pH 3, the samples buffered at pH 5 and 7.4 containing mixtures of **1** gave rise to another signal, *m*/*z* 326, assigned to the formation of **1a′** or **1a′′** adduct with two sodium ions. This may suggest that in the pH range of 5–7.4, the oxidation of **1** to **2** is not favoured regardless of the relatively high concentrations of hypochlorite (as postulated in Section 2.1). In the case of **2**, only the decarboxylation/chlorination step occurs, but the reaction product is identical to that derived from **1**. It is therefore clear that the hypochlorite recognition mechanism based on the oxidation of the carbonyl group in **1** with the formation of **2** is not the case, and that the main reason for fluorescence quenching in **1**–**3** upon their interaction with hypochlorite is the formation of non-fluorescent chlorine-substituted derivatives **1a′**–**3a′′**. No other products were detected.

### 2.4. Investigation into the Fluorescence of Probes in the Presence of Anti-Hypochlorite Agent Trolox

The hypochlorite-sensing ability of **1**–**3** was assessed in the presence of the anti-hypochlorite agent Trolox. Due to its well-known high antioxidative activity, Trolox is commonly applied as a standard for the determination of total antioxidative potential, which is also known as the Trolox Equivalent Antioxidant Capacity (TEAC) [44]. The testing was carried out on 150-μM solutions of probes **1**–**3** buffered at pH 3, 5, and 7.4, which were added into a solution containing 40 μM of Trolox and various concentrations of hypochlorite.

**Figure 5:**
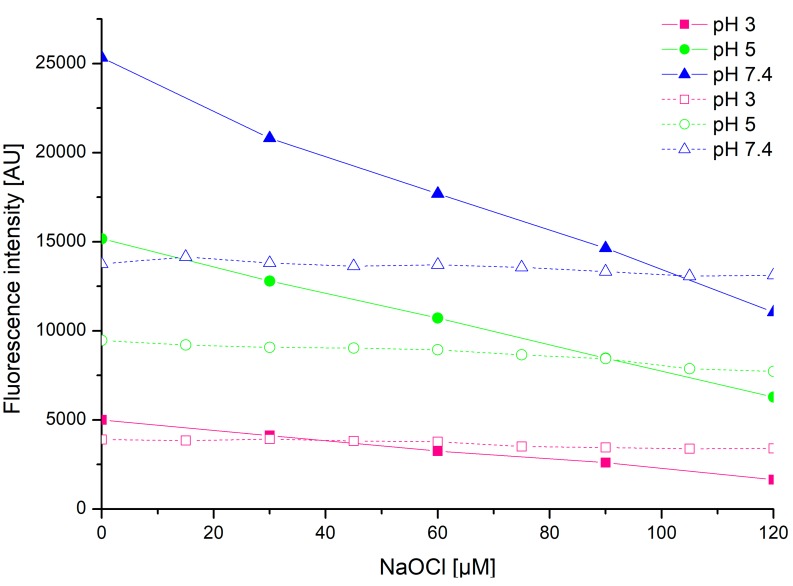
Comparison of sodium hypochlorite-induced changes in the fluorescence intensity of **2** (150 µM) (permanent lines), with that recorded upon the addition of Trolox (40 µM) (dashed lines). The measurements were carried out at λ_Ex_ 289 nm, λ_Em_ 460 nm, and at a temperature of 25 °C.

Probes **1**–**3** demonstrated a clear linear decrease in their fluorescence intensities upon increasing NaOCl concentration (Figure 2 and supplementary data), whilst the treatment of probe–hypochlorite mixtures with Trolox resulted in constant fluorescence intensities of the probes (Figure 5). This effect was clearly visible within 5 min of the addition of a probe into the Trolox–hypochlorite mixture and remained stable for the 30-min duration of the experiment (Figure 5, dashed lines). Further fluorescence intensity changes were not monitored. Although the fluorescence intensities of probes **1**–**3** remained unchanged within the whole range of the hypochlorite concentrations tested, they were notably lower compared to those recorded in the absence of Trolox (Figure 5). This effect was particularly clearly visible in mixtures buffered at pH 7.4. The differences are most likely due to the presence of additional components in the mixture, namely Trolox and products of its reaction with hypochlorite. Apparently, the presence of Trolox and its derivatives introduces a series of additional intermolecular interactions, which result in a decrease in the fluorescence quantum yields and thus in the fluorescence intensities [45] of probes **1**–**3**. Nevertheless, these environmental effects do not perturb the hypochlorite-sensing ability of probes **1**–**3** and their mode of action remains intact.

### 2.5. Assessment of Applicability of Probes 1–3 as Tools for Quantitative Determination of Hypochlorite

**Figure 6:**
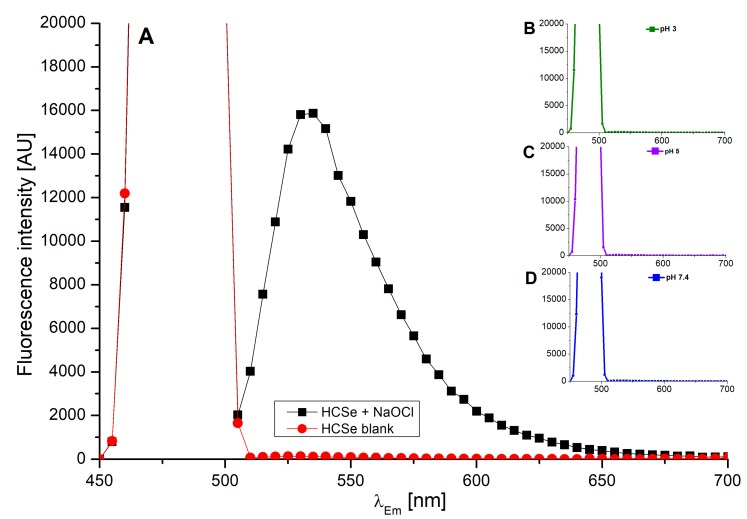
(**A**) Fluorescence intensity of 125 μM of HCSe probe in the presence of 150 μM of NaOCl at pH 5, 25 °C. Insets (**B**–**D**) Fluorescence of 150 μM of probe **2** under the influence of 150 μM of NaOCl and in the presence of 125 μM of HCSe probe at pH 3, 5, and 7.4 (λ_Ex_ 480 nm) at 25 °C.

An assessment of the applicability of probes **1**–**3** as tools for the quantitative determination of hypochlorite required evidence that their chlorination occurs with full conversion of hypochlorite. This was achieved by the treatment of 1:1 (probe:NaOCl) mixtures with a highly hypochlorite-specific turn-on fluorescent probe, namely HCSe [46]. No fluorescence emission was observed upon the addition of HCSe into the reaction mixtures, indicating a lack of unreacted hypochlorite (Figure 6).

Taking into account the linear dependence of the fluorescence intensity on the hypochlorite concentration (Figure 2) and the full hypochlorite conversion occurring with the formation of known derivatives (known recognition mechanism), a possible quantitative determination of the hypochlorite was considered based on the quantification of chlorinated products. The formation of chlorinated products of **1**–**3** is related to the hypochlorite content and hence its quantification may potentially be expressed as a function of percentage yields of chlorinated products formed. The HPLC-PDA-ESI-MS technique, which was utilised for the identification of the chlorinated products (Section 2.3) seems convenient for the hypochlorite quantification. In terms of potential use of the probes for the quantitative determination based on the derivatisation of the probes tested, probe **2** seems the most attractive. In detail, the highest conversion degree into the corresponding monochloro derivative **2a′** and the practically negligible formation of the additional isomer **2a′′** are among the advantages of this probe. Also, the dichloro-substituted derivative **1b** formation seems more straightforward, particularly when compared to that of probe **1**. Last but not least, the synthetic protocol for **2** is much easier than those for **1** and **3**, making access to this probe cost effective. Hence, further research on the potential practical application of **2** for the quantitative determination of hypochlorite is particularly warranted.

### 2.6. Isolation and Structural Characterisation of Chlorinated Derivative 2a′

**Figure 7:**
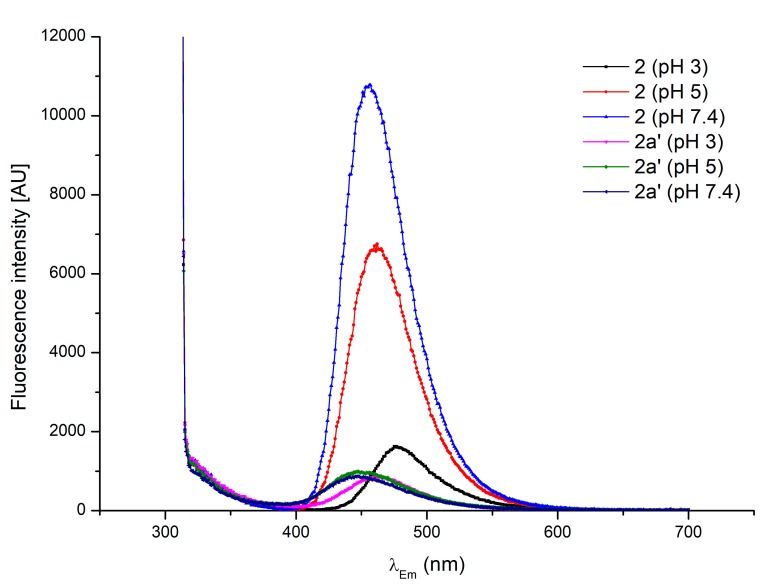
Steady-state fluorescence spectra of the isolated derivative **2a′** and its respective substrate **2** recorded at conditions identical to those applied for the hypochlorite sensing experiment (concentration of 150 μM, various pH levels, λ_Ex_ 289, temperature of 25 °C).

In order to prove that the chlorination reaction takes place, the main chlorinated derivative (**2a′**) was isolated by the treatment of **2** with excess sodium hypochlorite in acetate buffer. The product was obtained with a good yield and high purity. The LC-MS (retention time and *m*/*z* [M+H]^+^) results were consistent with these of proposed for **2a′** (Table 1). Under conditions identical to those applied for the hypochlorite sensing experiments, product **2a′** demonstrated a negligible fluorescence compared to that of **2** (Figure 7). Also, the comparison of FT-IR (ATR) spectra of substrate and the product demonstrated significant differences characteristic of the chlorinated derivative formation (Figure S10). 

## 3. Materials and Methods 

### 3.1. Materials

All chemicals used for the syntheses were of reagent grade or higher. 4-(diethylamino)salicylaldehyde, diethyl malonate, dimethylformamide, phospohorous oxychloride, acetic acid, piperidine, 7-diethylamino-4-methylcoumarin, Trolox, and MS-grade methanol were purchased from Aldrich (St. Louis, MO, USA). Concentrated HCl and solid NaOH were purchased from ChemPur (Piekary Śląskie, Poland). Ethanol, formic acid, sodium hypochlorite, and toluene were purchased from Avantor (Gliwice, Poland). All solvents were of 99% purity or higher (HPLC grade). The HCSe turn-on fluorescent probe was a kind donation from professors Shi-Rong Liu and Shu-Pao Wu from the Department of Applied Chemistry, National Chiao Tung University, Hsinchu, Taiwan, ROC.

### 3.2. Methods

The NMR spectra of coumarin derivatives were acquired on a Bruker Avance III spectrometer (500 MHz), using DMSO^−^d6 as a solvent. The infrared spectra were recorded in the region of 4000 cm^−1^ to 600 cm^−1^ on a Nicolet Impact 410 Fourier-Transform Infrared spectrophotometer equipped with an ATR adapter and Omnic software. Steady-state fluorescence measurements were performed in 96-well black plates (Nunc™ F96 MicroWell™ Black Polystyrene Plate, Thermo Scientific™, Roskilde, Denmark) on a Tecan Infinite 200 microplate reader (Tecan Austria GmbH, Grödig/Salzburg, Austria). HPLC-ESI-MS analyses were performed on an LCMS-8030 mass spectrometer (Shimadzu, Kyoto, Japan). All HPLC-MS analyses were performed in positive ion mode.

### 3.3. Synthesis of Coumarin Derivatives

Coumarin derivatives **1** and **2** were synthesised based on modified procedures that have been described previously [47,48].

#### 3.3.1. Synthesis of 7-Diethylamino-3-Formylcoumarin **1**

4-Diethylamino-2-hydroxybenzaldehyde (2.0 g, 10 mmol) and diethyl malonate (1.7 g, 10 mmol) were combined and piperidine (0.3 mL) was added. The mixture was stirred at room temperature for 3 h and the thick oil that formed was dissolved in concentrated HCl (15 mL) and glacial acetic acid (15 mL). The mixture was then heated under reflux and stirred for another 6 h. After cooling down to room temperature, the mixture was poured onto ice-cold water (60 mL) and the pH was carefully adjusted to 5 with saturated NaOH solution. The precipitate formed was then filtered off and washed with cold water and dried. The dry solid was then dissolved in toluene (20 mL), boiled, and hot-filtered to separate the dark solid. The dark-orange crystalline product, which formed upon cooling the toluene solution, was identified as 7-diethylaminecoumarin-3-carboxylic acid (**2**) and recovered by filtration (~0.2 g). The remaining solution was evaporated to dry the material under reduced pressure, yielding 0.61 g (54%) of 7-diethylaminocoumarin intermediate, which was then dissolved in DMF (5 mL).

POCl_3_ (1 mL) was placed in round-bottom flask and stirred at 50–60 °C under an inert atmosphere, while DMF (1 mL) was added dropwise. After approximately 30 min of heating and stirring, the mixture changed its colour to pale red, and then the solution of 7-diethylaminecoumarin in DMF was added. The mixture was stirred at 60 °C for 18 h, then cooled and poured onto ice-cold water (50 mL). The pH was adjusted to approximately 7.5 by the dropwise addition of saturated NaOH solution. The fine solid was filtered off, washed with a small amount of cold water, and dried in air, yielding 7-diethylamino-3-formylcoumarin (**1**). Yield 0.4 g (31%); C_14_H_15_NO_3_ (245.11 g/mol); ^1^H-NMR (DMSO): δ = 9.90 ppm (s, 1H, H3 (-CHO)), 8.41 (s, 1H, H4), 7.68 (d, 1H, H5, *J* = 9.05 Hz), 6.83 (dd, 1H, H6, *J_1_* = 9.05 Hz, *J_2_* = 2.38 Hz), 6.60 (d, 1H, H8 *J* = 2.38 Hz), 3.51 (q, 4H, (-CH_2_-)), 1.15 (t, 6H, (-CH_3_)); *m*/*z* [M+H]^+^ 246.05.

#### 3.3.2. Synthesis of 7-Diethylaminocoumarin 3-Carboxylic Acid **2**

4-Diethylamino-2-hydroxybenzaldehyde (1.0 g, 5 mmol) and diethyl malonate (0.83 g, 5 mmol) were combined in a round-bottom flask and piperidine (0.15 mL) was added. The mixture was stirred at room temperature for 3 h, after which water (10 mL) was added. The pH was then adjusted to 5 using the diluted HCl and the thick oil that formed was separated and washed with cold water. The oily product, identified as ethyl 7-diethylaminocouamrin-3-carboxylate, was dissolved in 25 mL of ethanol/water (2:1 *v*/*v*) and then solid NaOH (1.5 g, 37 mmol) was added. The mixture was heated to reflux for 30 min and poured onto an ice-cold solution of 20% HCl (10 mL), cooled down to the ambient temperature, and refrigerated for another 30 min. The dark-orange precipitate was filtered off, washed with cold water, and recrystallised from ethanol to yield 7-diethylaminocoumarin 3-carboxylic acid (**2**). Yield 0.86g (64%); C_14_H_15_NO_4_ (261.28 g/mol); ^1^H-NMR (DMSO): δ = 12.48 ppm (s, 1H, H3 (-COOH)), 8.58 (s, 1H, H4), 7.64 (d, 1H, H5, *J* = 9.04 Hz), 6.80 (dd, 1H, H6, *J_1_* = 9.04 Hz, *J_2_* = 2.30 Hz), 6.57 (d, 1H, H8 *J* = 2.30 Hz), 3.49 (q, 4H, (-CH_2_-)), 1.14 (t, 6H, (-CH_3_)); *m*/*z* [M+H]^+^ 262.00.

#### 3.3.3. Isolation of Chlorinated Derivative of 7-Diethylaminocoumarin 3-Carboxylic Acid **2a′**

A suspension of **2** in acetate buffer (pH 5.0) was treated with sodium hypochlorite and stirred at room temperature until the flocculent precipitate appeared. The mixture was then treated with ethanol, followed by further addition of sodium hypochlorite until a yellow coloration of the mixture appeared. The extraction with chloroform followed by evaporation under reduced pressure yielded the orange precipitate identified as **2a′**. Yield 0.36 g (58%); *m*/*z* [M+H]^+^ 295.95; UV-Vis (methanol) λ_max_ 369 nm; Fluorescence (methanol) λ_Ex_ 289 nm, λ_Em_ 441 nm; IR(ATR): 2978, 1740, 1667, 1586, 1505, 1574, 1202, 1094, 796, 662, 476. 

### 3.4. Fluorescence Assay

First, 1 mM probe solutions in ethanol were subjected to the influence of varying concentrations of NaOCl. Given that the chlorine concentration in saline is approximately 150 μM, we assumed a 1:1 stoichiometric ratio of the probe to chlorine; thus, in all experiments the probe concentration was set at 150 μM per sample.

#### 3.4.1. Determination of the Effect of Hypochlorite on the Emission Properties of the Probes

In this step, 30 μL of 1 mM probe solution was applied to a black, 96-well plate. Samples were buffered with 20 μL of 25 mM acetate (pH 3 and 5) or phosphate (pH 7.4) buffers. Next, an increasing concentration of NaOCl was added so that its final amount in the total volume (200 μL) of samples was in the range of 0 to 230 μM. The plate was shaken for 10 s on the reader shaker and then the fluorescence measurement was started. All samples were excited by light at a wavelength of λ_Exc_ 289 nm and the fluorescence spectra at λ 300–650 nm were recorded at 25 °C.

#### 3.4.2. Investigation into the Fluorescence of Probes in the Presence of Anti-Hypochlorite Agent

To a black, 96-well plate, 20 μL of 25 mM acetate (pH 3 and 5) or phosphate (pH 7.4) buffers and increasing concentrations of NaOCl was added. The final concertation of hypochlorite in the total volume (200 μL) of samples was in the range of 0 to 120 μM. Next, 80 μL of 0.1 mM Trolox solution was applied and after 5 min of reaction 30 μL of 1 mM probe solution was added. The plate was shaken for 10 s on the reader shaker and then the fluorescence measurement was started. All samples were excited by light at a wavelength of λ_Exc_ 289 nm and the fluorescence spectra at λ 300–650 nm were recorded at 25 °C. 

### 3.5. Mass Spectrometry Analysis

The mass spectrometer was controlled by LabSolutions software (Shimadzu), the electrospray voltage was set at 4.5 kV, the capillary temperature was at 170 °C, and N_2_ was used as a sheath gas. Samples were eluted through 150 mm × 4.6 mm i.d., 5-μm Kinetex C18 column (Phenomenex) protected by a 4 mm × 2 mm i.d. guard column of the same material. The injection volume was 2 μL, the flow rate was 0.5 mL/min, and the column was thermostated at 40 °C. The HPLC solvents gradient was 50% B in A at 0 min to 80% B in A at 12 min (A: 2% *v*/*v* formic acid in water; B: methanol). UV-Vis spectra were recorded using a photodiode array detector (PDA) (Shimadzu, Kyoto, Japan). The LC-MS protocol applied was developed based on previous studies [49,50,51,52]. The low aqueous solubility of all compounds tested necessitated the application of a relatively strong gradient.

## 4. Conclusions

In conclusion, we have investigated three highly fluorescent 7-diethylamine-substituted coumarin derivatives (**1**–**3**) for their hypochlorite-sensing potency. Based on the set of spectroscopic and chromatographic experiments, we have elucidated the hypochlorite recognition mechanism for all probes tested. The interaction of probes **1**–**3** with sodium hypochlorite leads to the formation of non-fluorescent chlorinated derivatives, which points indicates that the chlorination reaction is responsible for the linear fluorescence decays of **1**–**3**. No similar behaviour of hypochlorite-sensitive coumarins has been reported to date. The structures of chlorinated products were proposed and their percentage yields were determined based on LC-MS analysis. Taking into account the previously reported promising selectivity of such compounds [34,47] the results obtained herein give strong evidence for the applicability of **1**–**3** as potential hypochlorite markers and indeed the results raise the possibility of using compounds **1**–**3** for the quantitative determination of ClO^−^. In this context, the relatively cheap and straightforward synthesis of probe **2** makes this compound particularly attractive. Additionally, it is worth emphasizing that the formation of coumarin derivatives chlorinated at the lactone ring is non-trivial and usually requires harsh conditions. Therefore, the successful isolation of chlorinated derivative **2a′** gives access to a new series of chlorinated coumarins obtained at relatively mild conditions, which in turn opens new possibilities for further modifications of the coumarin nucleus.

## Figures and Tables

**Table 1:** Chromatographic, spectrophotometric, and mass spectrometric data for the coumarin derivatives **1**–**3** and their corresponding chlorinated products at pH 3 after 15 min of reaction with hypochlorite.

Compound No.	Compound Name	t_ret_ (min)	λ_max_ (nm)	*m*/*z* [M+H]^+^	Composition (%)
**1**	7-diethylamino-3-formylcoumarin	7.9	443	246.05	29.1
**1a′**	Monochloro-7-diethylamino-3-formylcoumarin ^*^	9.7	433	279.95	25.8
**1a′′**	monochloro-7-diethylamino-3-formylcoumarin ^*^	11.1	440	279.05	2.5
**1b**	dichloro-7-diethylaminocoumarin ^*^	12.1	366	285.95	4.2
**2**	7-diethylaminocoumarin-3-carboxylic acid	8.1	432	262.00	15.6
**2a′**	monochloro-7-diethylaminocoumarin-3-carboxylic acid ^*^	9.9	411	295.95	33.5
**2a′′**	monochloro-7-diethylaminocoumarin-3-carboxylic acid ^*^	10.9	396	295.95	0.5
**1b**	dichloro-7-diethylaminocoumarin ^*^	12.1	366	285.90	5.9
**3a′**	monochloro-7-diethylamino-4-methylcoumarin ^*^	10.2	350	266.00	44.3
**3**	7-diethylamino-4-methylcoumarin	10.5	375	232.05	27.2
**3a′′**	monochloro-7-diethylamino-4-methylcoumarin ^*^	12.9	389	266.00	19.1
**3b**	dichloro-7-diethylamino-4-methylcoumarin ^*^	13.3	360	299.95	6.1

^*^ Proposed product.

## Supplementary Materials

Supplementary materials can be found at http://www.mdpi.com/1422-0067/20/2/281/s1.

## Abbreviations

ICT	intramolecular charge transfer
PET	photO^−^induced electron transfer
FRET	fluorescence resonance energy transfer
ESIPT	excited state intramolecular proton transfer
ROS	reactive oxygen species
HCSe	boron-dipyrromethene-based turn-on fluorescent probe
ClO^−^	hypochlorite ion
PDA	photodiode array
ESI	electrospray ionisation
