# Supplementary material for: Fluorescence Quenching-Based Mechanism for Determination of Hypochlorite by Coumarin-Derived Sensors
Source: Int J Mol Sci. 2019 Jan 11;20(2):281. doi: 10.3390/ijms20020281 (PMC6358793; doi:10.3390/ijms20020281)
Supplement: Supplementary file 1 [file ijms-20-00281-s001.pdf]

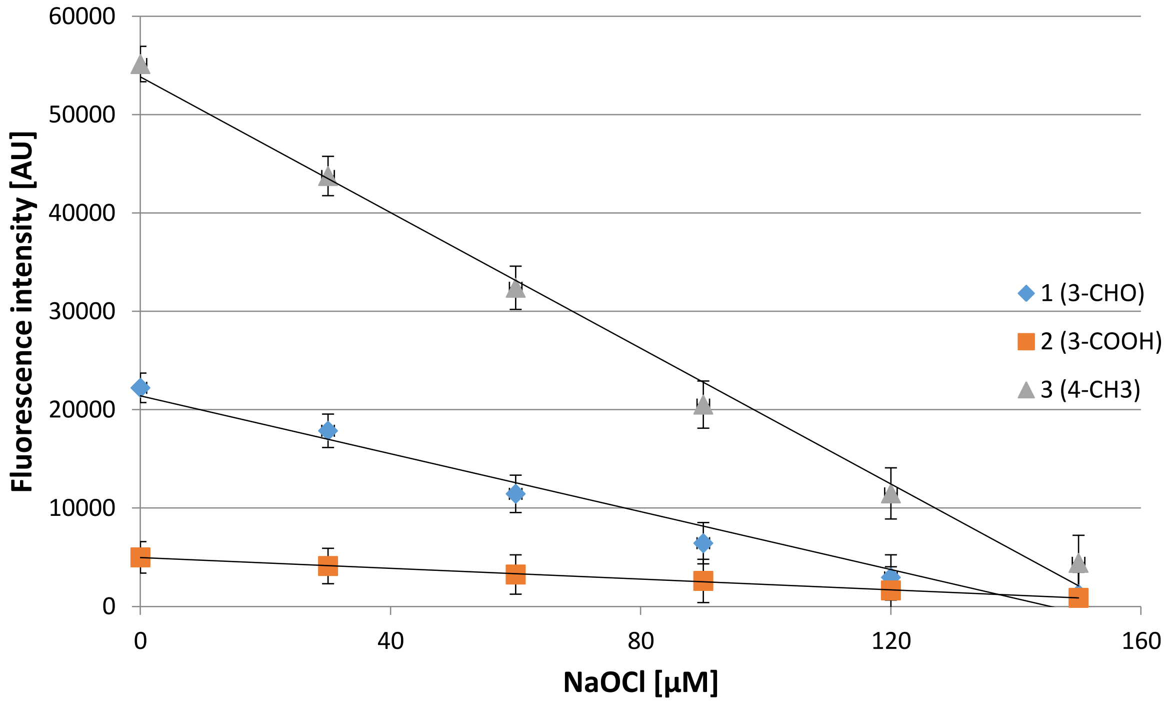

Figure 1S. Changes in fluorescence intensity of **1- 3** induced by an increase in NaOCl concentration, at pH 3, λ_{Ex} 289 nm, and the temperature of 25 °C.

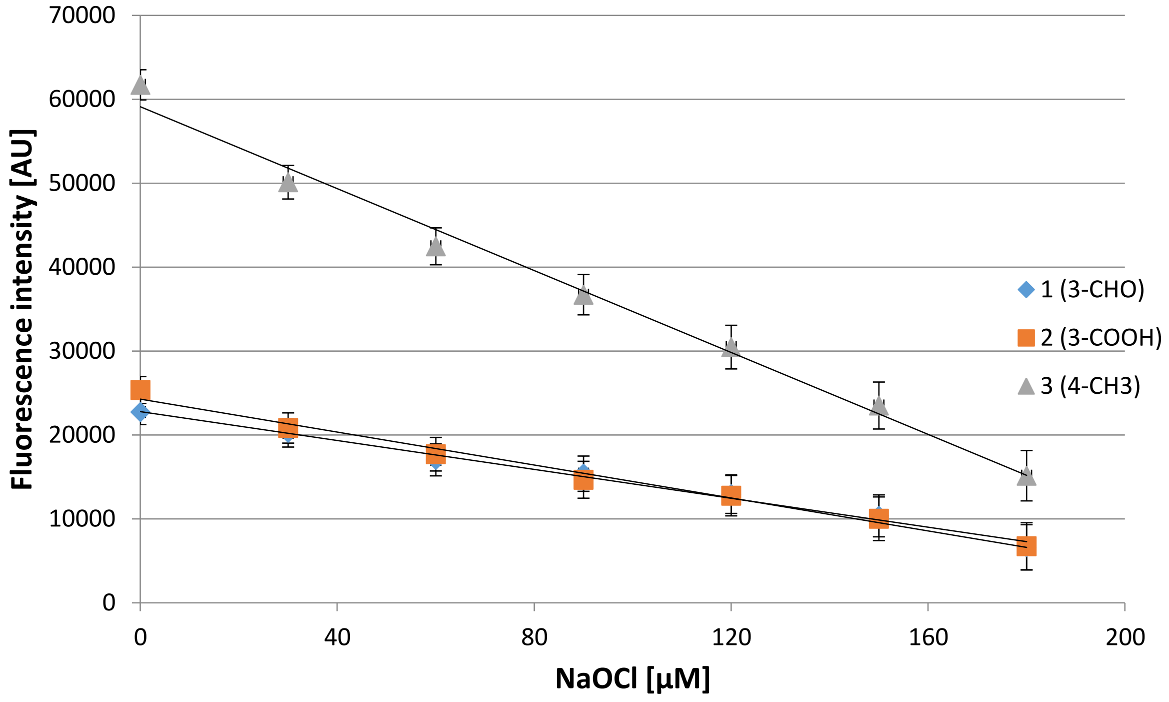

Figure 2S. Changes in fluorescence intensity of **1- 3** induced by an increase in NaOCl concentration, at pH 7.4, λ_{Ex} 289 nm, and the temperature of 25 °C.

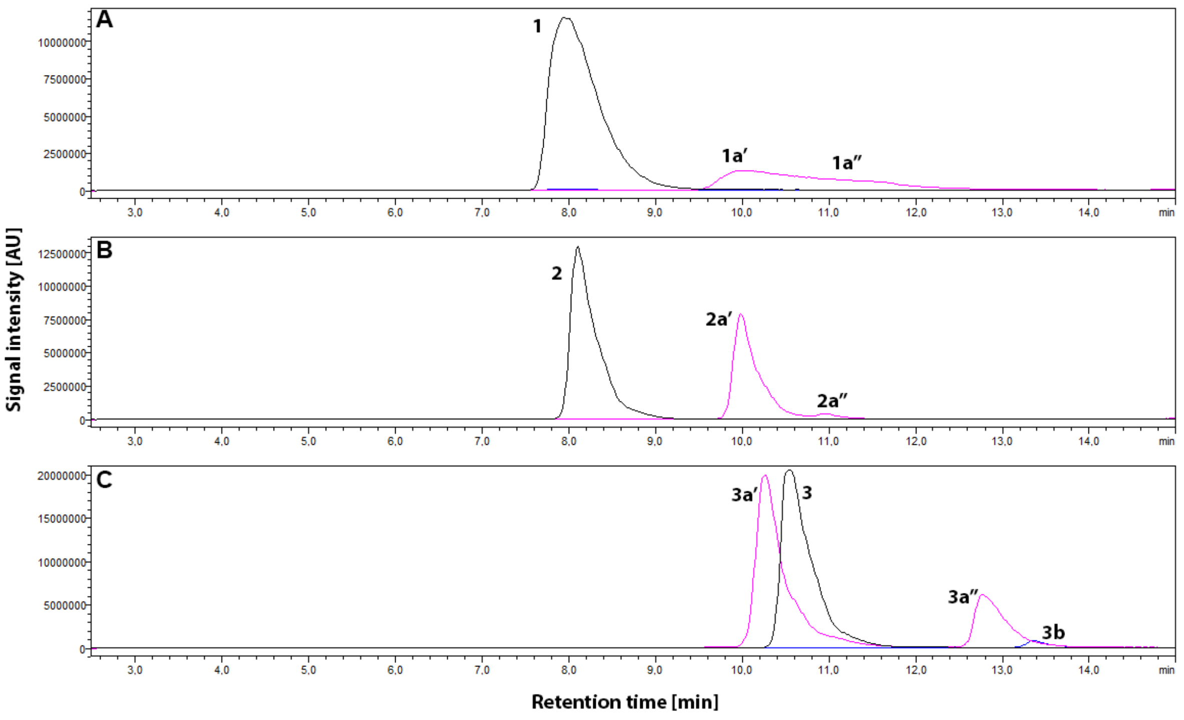

Figure 3S. HPLC-MS traces of chlorination products of 150 µM probes **1** (A), **2** (B), **3** (C) by 150 µM NaOCl at pH 5 and the temperature of 25 °C. Peak numbering is presented in Table 1.

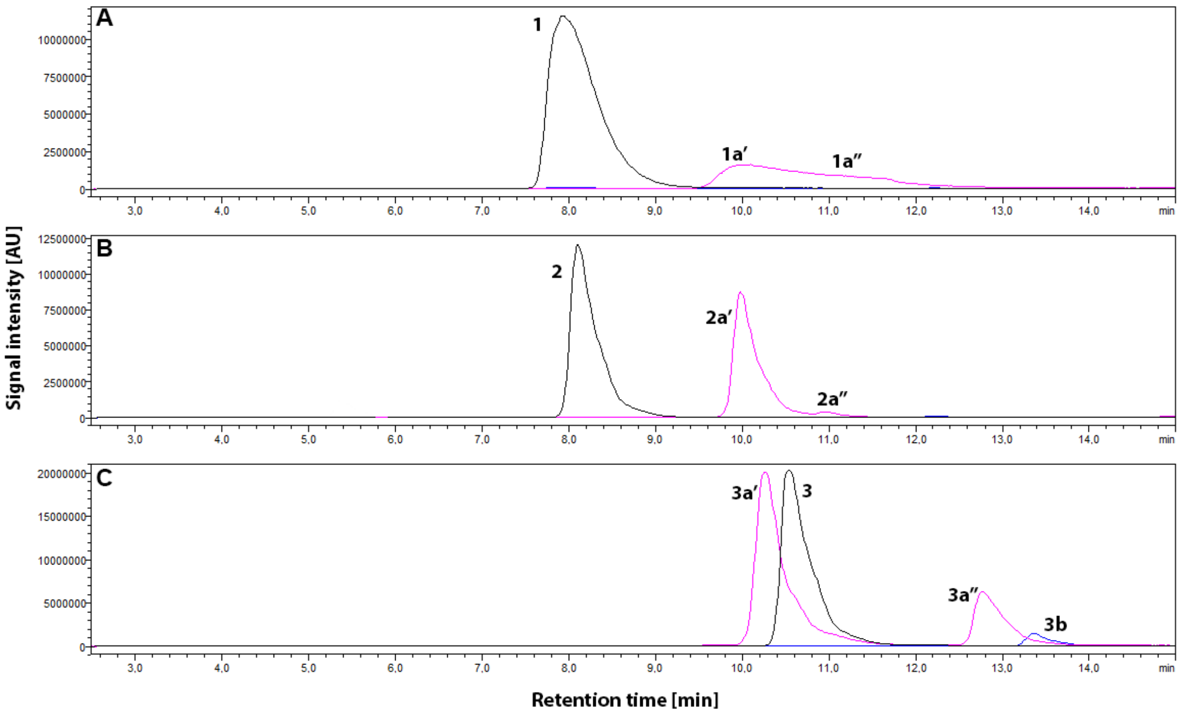

Figure 4S. HPLC-MS traces of chlorination products of 150 µM probes **1** (A), **2** (B), **3** (C) by 150 µM NaOCl at pH 7.4 and the temperature of 25 °C. Peak numbering is presented in Table 1.

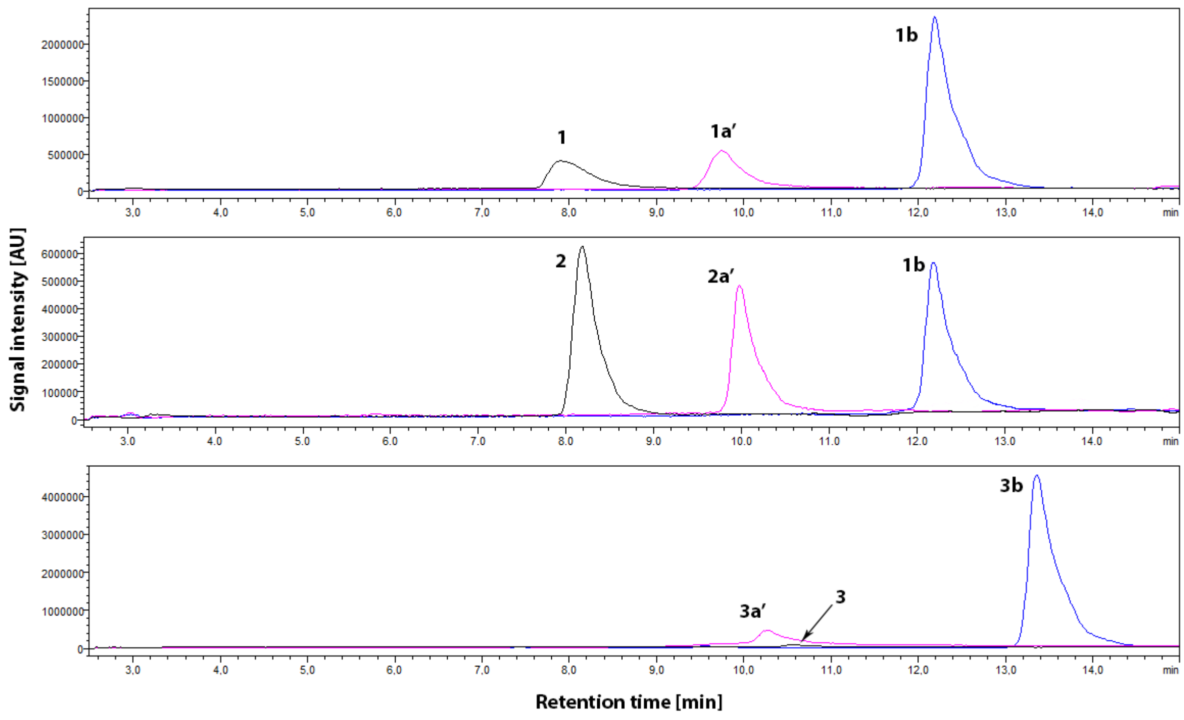

Figure 5S. HPLC-MS traces of chlorination products of 150 μM probes **1-3** by 5-fold excess NaOCl at pH 3 and the temperature of 25 °C. Peak numbering is presented in Table 1S.

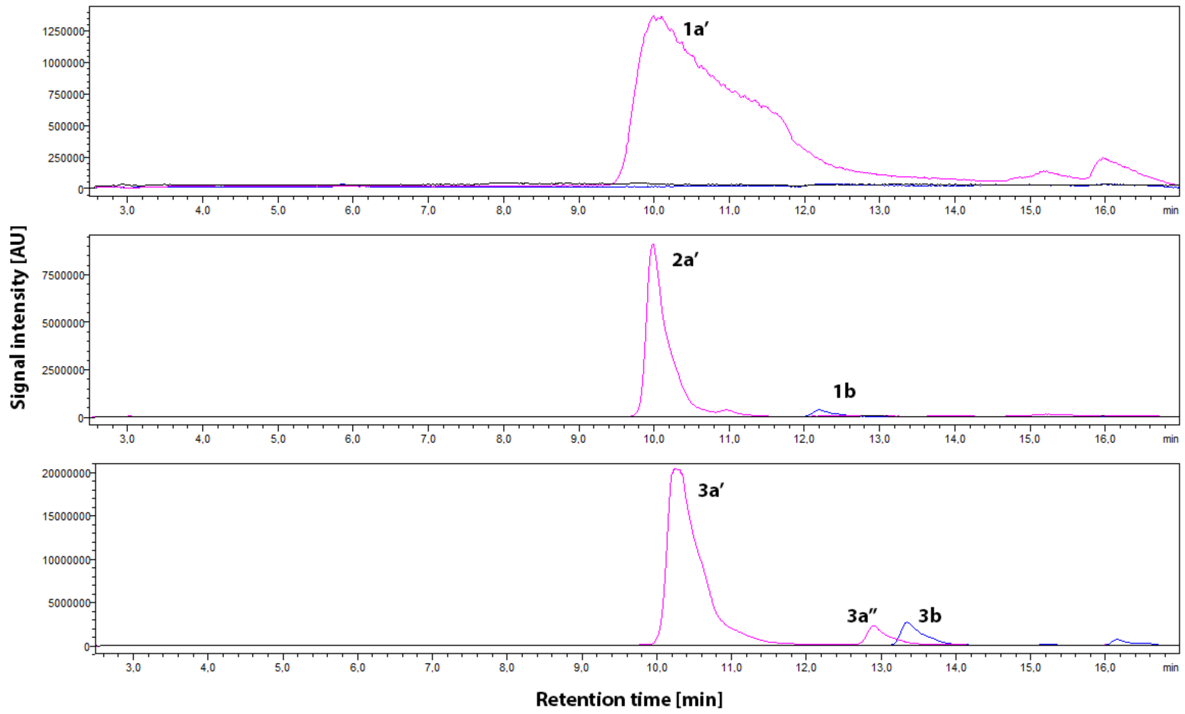

Figure 6S. HPLC-MS traces of chlorination products of 150 μM probes **1-3** by 5-fold excess NaOCl at pH 5 and the temperature of 25 °C. Peak numbering is presented in Table 1S.

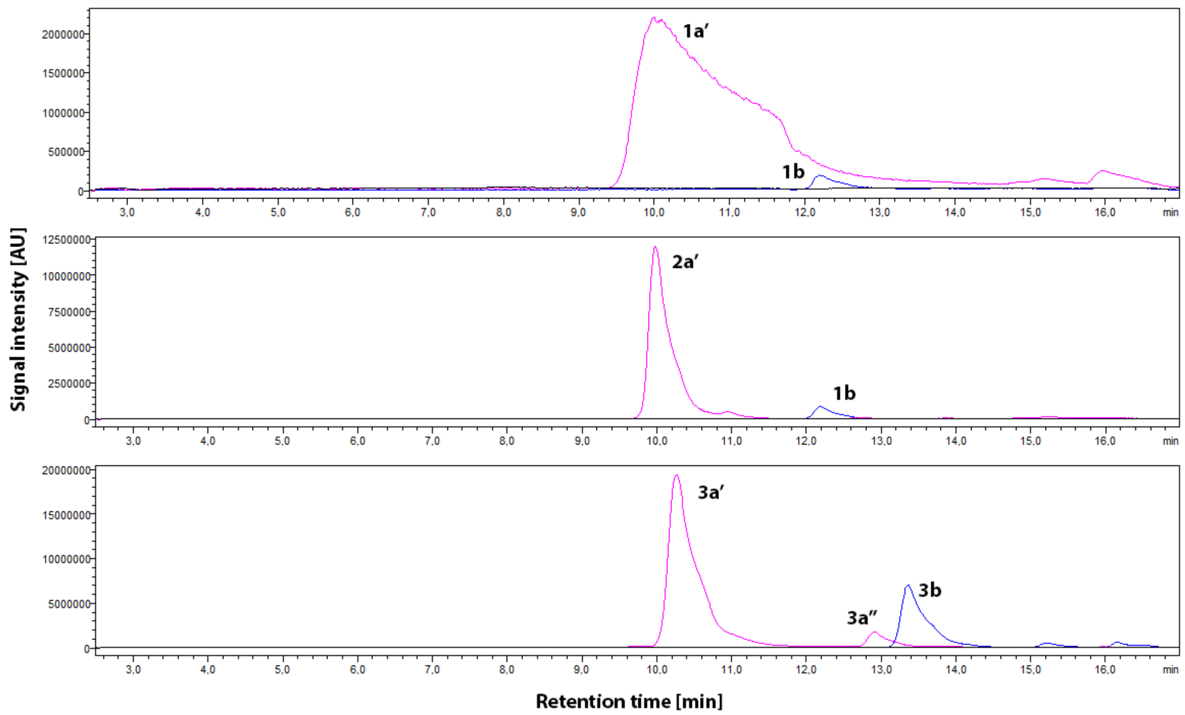

Figure 7S. HPLC-MS traces of chlorination products of 150 μM probes **1-3** by 5-fold excess NaOCl at pH 7.4 and the temperature of 25 °C. Peak numbering is presented in Table 1S.

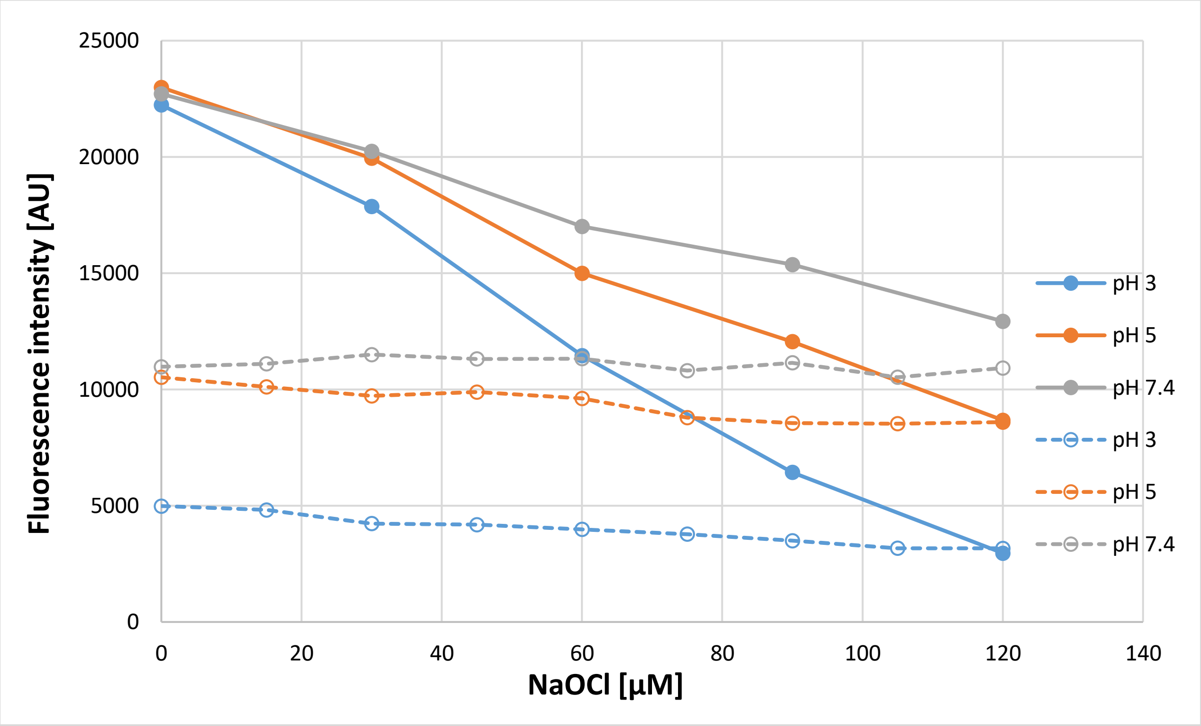

Figure 8S. Comparison of sodium hypochlorite-induced changes in fluorescence intensity of **1** (150 μM) (permanent lines), with that recorded upon the addition of Trolox (40 μM) (dashed lines). The measurements were carried out at λ_{Ex} 289 nm, λ_{Em} 464 nm, and a temperature of 25 °C.

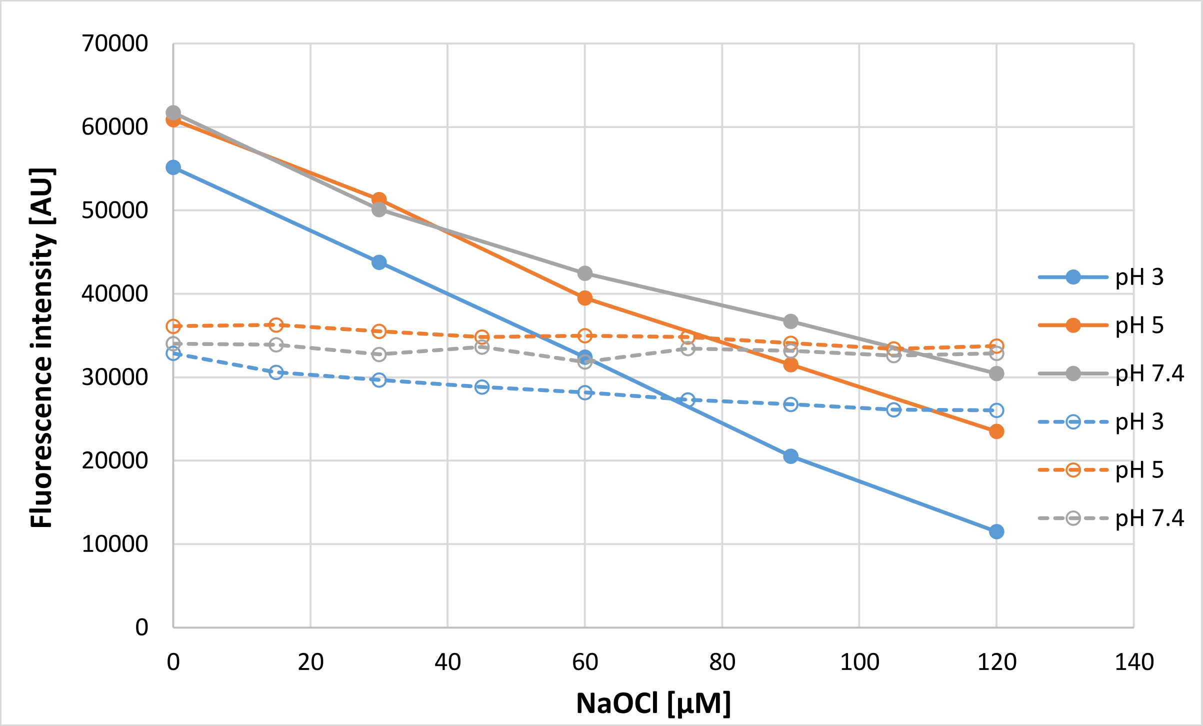

Figure 9S. Comparison of sodium hypochlorite-induced changes in fluorescence intensity of **3** (150 µM) (permanent lines), with that recorded upon the addition of Trolox (40 µM) (dashed lines). The measurements were carried out at λ_{Ex} 289 nm, λ_{Em} 450 nm, and a temperature of 25 °C.

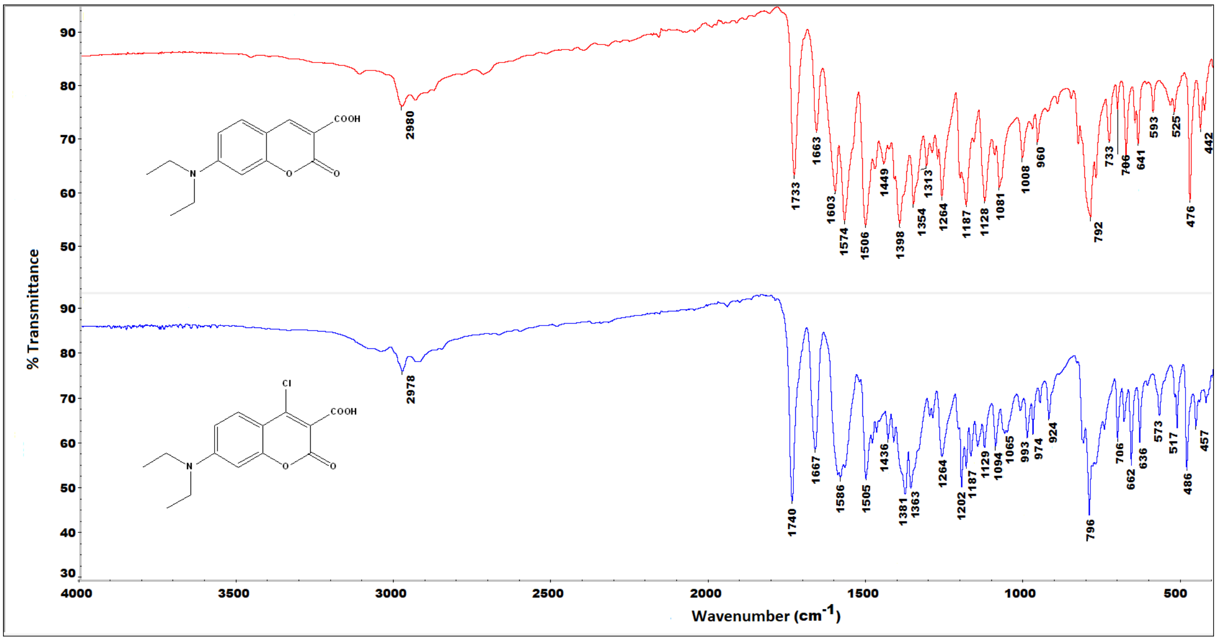

Figure 10S. Comparison of IR(ATR) Spectra of **2** (upper) and its corresponding chlorinated derivative **2a'** (bottom)

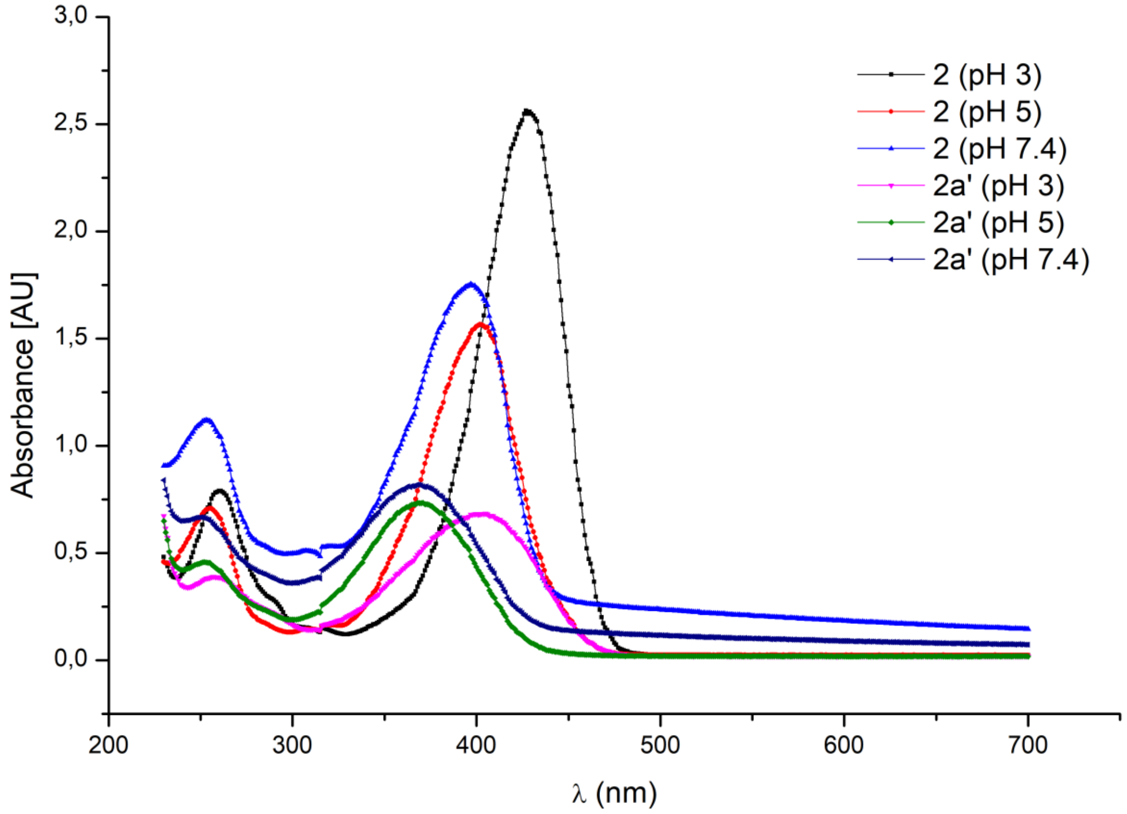

Figure 11S. UV-Vis absorption spectra of the isolated derivative **2a'** and its respective substrate **2** recorded at conditions identical to those applied for the hypochlorite sensing experiment (concentration of 150 uM, various pH, λ_{Ex}=289, and the temperature of 25 °C).

Table 1S. Chromatographic, spectrophotometric, and mass spectrometric data for the coumarin derivatives **1-3** and their corresponding chlorinated products at pH 3 after 15 minutes of reaction with fivefold excess of hypochlorite.

**Compound No.**	**Compound name**	**t_{ret} [min]**	**λ_{max} [nm]**	***m/z* [M+H]$^+$**	**Composition [%]**
**1**	7-diethylamino-3-formylcoumarin	7.9	443	246.05	1.6
**1a′**	monochloro-7-diethylamino-3-formylcoumarin*	9.7	433	279.95	2.0
**1b**	dichloro-7-diethylaminocoumarin*	12.1	366	285.95	8.7
**2**	7-diethylaminocoumarin-3-carboxylic acid	8.1	432	262.00	3.0
**2a′**	monochloro-7-diethylaminocoumarin-3-carboxylic acid*	9.9	411	295.95	2.1
**1b**	dichloro-7-diethylaminocoumarin*	12.1	366	285.90	2.6
**3a′**	monochloro-7-diethylamino-4-methylcoumarin*	10.2	350	266.00	0.7
**3**	7-diethylamino-4-methylcoumarin	10.5	375	232.05	0.1
**3b**	dichloro-7-diethylamino-4-methylcoumarin*	13.3	360	299.95	9.8
